# Changes in recreational behaviors of outdoor enthusiasts during the COVID-19 pandemic: analysis across urban and rural communities

**DOI:** 10.1093/jue/juaa020

**Published:** 2020-08-14

**Authors:** William L Rice, Timothy J Mateer, Nathan Reigner, Peter Newman, Ben Lawhon, B Derrick Taff

**Affiliations:** j1 Department of Society and Conservation, University of Montana, 32 Campus Drive, Missoula, MT 59812, USA; j2 Department of Recreation, Park, and Tourism Management, Pennsylvania State University, 801 Ford Building, University Park, PA 16802, USA; j3 Leave No Trace Center for Outdoor Ethics, 1000 North St, Boulder, CO 80304, USA

**Keywords:** COVID-19, coronavirus, pandemic, outdoor recreation, recreation planning, urban outdoor recreation

## Abstract

The COVID-19 pandemic presents not only a global health crisis but has also disrupted the daily lives of people around the world. From a leisure perspective, urban outdoor enthusiasts are one group particularly impacted by the pandemic and the subsequent institutional response. Stay-at-home orders and physical distancing recommendations serve as potential inhibitors to outdoor recreation activities central to the lifestyles and wellbeing of outdoor enthusiasts. In urban areas, where these orders and recommendations are most restrictive, the potential impacts on recreation behavior are most consequential. This study provides an empirical analysis of the COVID-19 pandemic’s impact on the recreational behaviors of outdoor enthusiasts across urban and rural communities. Results suggest that the frequency of outdoor recreation participation, distance travelled to participate in outdoor recreation and distance travelled beyond roads during outdoor recreation have declined significantly more among outdoor enthusiasts residing in urban areas than urban clusters or rural areas.

## Introduction

Outdoor recreation provides important recreational ecosystem services (e.g. stress relief, socialization, nature appreciation, etc.) through physical interaction with the natural world (Scholte et al. 2019). In times of crisis or disaster, outdoor recreation also provides an important means of coping (Rung et al. 2011; Samuelsson et al. 2020). The COVID-19 pandemic presents an unprecedented global health crisis (Stier, Berman and Bettencourt 2020). Limitations to travel across all scales—from intercontinental tourism to stay-at-home orders—have brought significant stress to the global community (Bao et al. 2020). Though necessary to thwart the spread of the virus, diminished or restricted access to settings that facilitate outdoor recreation reduce a community’s capacity to cope with crisis, especially for the outdoor enthusiast subculture—those who are highly dependent on outdoor recreation as a means of leisure (see Outdoor Industry Association, 2015). Such necessary restrictions to access have also complicated the role parks and outdoor recreation play in the promotion of psychological and physiological recovery during crisis (Rung et al. 2011; Samuelsson et al. 2020). Loss of access to outdoor recreation opportunities inhibits individuals’ abilities to engage with restorative natural environments and escape the pressures of the crisis (Rung et al. 2011; Samuelsson et al. 2020). It is therefore imperative that officials and planners have access to information concerning changes to outdoor recreation behaviors (De Valck et al. 2016).

### The impact of the COVID-19 pandemic on recreation access

The World Health Organization (WHO) officially declared the coronavirus disease 2019 (COVID-19) as a pandemic on 11 March 2020. In the organization’s official announcement, the WHO Director-General called on all countries to ‘detect, test, treat, isolate, trace, and mobilize their people in the response…[and] reduce transmission’ (WHO 2020). In response, many countries issued restrictions to travel outside of the home to reduce the transmission of the highly contagious virus (Tufan and Kayaaslan 2020). Scotland, for example, explicitly prohibited non-local travel for recreation and leisure purposes (Scottish Government 2020). As demonstrated by Stier, Berman and Bettencourt (2020), the spread of COVID-19 intensifies with city size—meaning that larger urban areas experience faster, more devastating outbreaks. Therefore, cities have moved to implement strict physical distancing measures and limitations to public green space (Samuelsson et al. 2020). It is posited that these restrictions to outdoor recreation—though necessary for controlling the spread of COVID-19—have unintended negative consequences on the wellbeing of urban populations, as ‘access to urban nature is especially important when stress levels are high in populations that suddenly are asked to shelter in place and that experience anxiety due to uncertainty and fear of infection’ (Samuelsson et al. 2020, p. 2).

### Study purpose

Beyond the context of the present crisis, outdoor recreation has also been robustly linked to physical, mental and social health and wellbeing through the provisioning of ecosystem services (see reviews by Thomsen, Powell and Allen 2013; Holland et al. 2018). In urban areas, the provisioning of these ecosystem services can be enhanced through design and planning that increase exposure to natural features in recreation settings (Tan et al. 2020). In turn, it is important to understand how recreation patterns are shifting in response to COVID-19 and how the provisioning of recreational ecosystem services may be impacted so that improved park management, planning, and design can be implemented (Samuelsson et al. 2020). As noted by Salama (2020), this research is especially important for urban residents—who have lesser outdoor recreation amenities at their disposal and relatively higher place identity or dependence—as it is posited that the pandemic may have significant impacts on their mobility patterns.

Given the importance of outdoor recreation for certain groups (i.e. outdoor enthusiasts) in coping with crisis and the restrictions on travel placed in response to COVID-19 pandemic, it is imperative that officials and planners understand how the pandemic is impacting outdoor enthusiasts—those who are highly reliant on outdoor recreation as a means of leisure (Salama 2020). Outdoor enthusiasts represent a subculture that makes up a significant segment of the consumer marketplace and include those who ‘love being outdoors’ through a variety of recreational mediums (Outdoor Industry Association 2015). As identified by Salama (2020) and Samuelsson et al. (2020), the recreational impacts of the COVID-19 pandemic are appropriate for immediate study. Additionally, given the findings of Stier, Berman and Bettencourt (2020) concerning the spread of COVID-19 across urban and rural communities, it is imperative to understand what types of communities are most impacted by the pandemic in terms of their outdoor recreation behaviors. Therefore, the goal of this study is to understand how the COVID-19 pandemic impacts the recreation behaviors of outdoor enthusiasts across the community classifications of the urban–rural continuum.

## Methods

### Survey distribution

The online community of the Leave No Trace (LNT) Center for Outdoor Ethics was selected as the respondent pool for this research due to the frequency with which its members pursue outdoor recreation. LNT is a prominent outdoor education entity present in U.S. parks and protected areas, with a storied history, and established memorandums of understanding with all the federal land management agencies and many local and state park systems (Marion 2014). Its members are primarily American outdoor enthusiasts who spend 8–12 hours engaged in outdoor recreation (i.e. hiking, biking, camping, running, wildlife viewing, etc.) each week (LNT 2018). A Qualtrics-based online survey concerning recreation patterns during the COVID-19 pandemic was distributed to 63,890 subscribers of the LNT email listserv. Given the temporally dynamic nature of the COVID-19 pandemic, the survey was available for only a 48-hour window beginning on 9th April at 9 AM Mountain Standard Time (USA). Potential respondents were asked to participate in a 10- to 12-minute survey collecting relevant data on recreation patterns.

### Survey development

Given the study’s purpose, survey items were developed to collect data concerning how outdoor recreation behaviors are changing. Behaviors of interest included frequency of outdoor recreation, outdoor recreation group size, distance travelled to participate in outdoor recreation and distance travelled beyond roads during outdoor recreation. Each of these behaviors was selected to represent a different aspect of the recreational experience that has been linked to physical, mental or social wellbeing (Holland et al. 2018). For each behavior, changes in recreation patterns were measured for both the month before and the weeks after 11 March 2020—the date the World Health Organization declared the COVID-19 pandemic. Groups of interest across the rural–urban continuum were developed using community classification measures established by the U.S. Census Bureau, including those residing in rural areas with populations less than 5000, those residing in urban clusters with populations between 5000 and 50,000, and those residing in urbanized areas with populations exceeding 50,000 (U.S. Census Bureau 2010). Methods of measurement for behaviors and groups of interest are detailed in Table 1.

**Table 1:** Summary of behaviors and groups of interest

Behaviors of interest^a^	
Behaviors	Measurement of behaviors
Average frequency of outdoor recreation participation	Two single answer, multiple choice questions: <1 day to 7 days per week
*How many days per week did you participate in outdoor recreation for each of the following time windows before and after 11th March 2020?*	
Average outdoor recreation group size	Two numeric text-based free response questions
*What is [or was] the typical group size with which you participate[d] in outdoor recreation for the month prior to 11th March and since 11th March?*	
Average distance travelled to participate in outdoor recreation	Two single answer, multiple choice questions: 0–2 miles, 3–5 miles, 6–15 miles, 16–50 miles, or greater than 50 miles
*Approximately how far do [or did] you travel, on average, to participate in outdoor recreation for the month prior to 11th March and since 11th March?*	
Average miles travelled beyond roads during outdoor recreation	Two slider-based free response questions, where distances are represented to the precision of one decimal place
*Approximately how far from a road do [or did] you venture, on average, during your outdoor recreation activities for the month prior to 11th March and since March 11th?*	

Groups of interests	
Groups	Measurement of groups
Residents of communities across the rural–urban continuum	Reported type of community of residence adapted from the U.S. Census Bureau (2010): under 5000, between 5000 and 50 000, and over 50 000 residents

a All behavior-related questions were explicitly asked in relation to the COVID-19 pandemic.

### Data analysis

A series of one-way analyses of variance (ANOVA) were used to analyze differences in outdoor recreation behavior change among the three community classifications (rural area, urban cluster and urban area). One-way ANOVAs are used to determine differences in means for measured continuous variables across groups within a categorical independent variable (Vaske 2008). A series of one-way ANOVAs were used in lieu of a single MANOVA based on the guidance of Huberty and Morris (1989). For each ANOVA, Levene’s *F* test was used to determine if equality of variance could be assumed for behaviors. If equality of variance could not be assumed, Welch’s test of Equality of Means was used to correct the significance level of the omnibus test. Additionally, post hoc tests were conducted to assess significant differences among each of the three groups. The post hoc test used was determined by whether equality of variance could be assumed (Scheffé’s S or Tamhane’s T2). These post hoc tests were selected as they offer conservative estimates of significance (Vaske 2008).

## Results

Of the 63,890 total recipients of the distribution email, 3,003 individuals opened the email containing the survey link. In total, 1012 respondents agreed to complete the survey for a gross response rate of 4.7% or an adjusted response rate of 33.7% (based on opened emails)—within the expected range for online surveys (Blumenberg and Barros 2018). Respondents resided across 49 US states and territories and 14 countries, consisted of 57.8% females, and had an average age of 47 years old. Full descriptive results of the sample can be found in Table 2.

**Table 2:** Descriptive results of the sample

Group	*n*	Percent of sample
Gender		
Female	540	57.8%
Male	364	39.0%
Transgender	2	0.2%
Non-binary/Other	14	1.5%
Ethnicity		
White	825	88.5%
Hispanic or Latina/Latino/Latinx	30	3.2%
Asian or Pacific Islander	20	2.1%
Black or African American	7	0.8%
Native American, American Indian or Alaska Native	6	0.6%
Other	15	1.6%
Country of residence		
US resident	913	97.4%
Non-US resident	24	2.6%
Community classification of residence		
Rural (population < 5000)	324	34.7%
Urban cluster (population between 5000 and 50 000)	231	24.7%
Urbanized area (population > 50 000)	380	40.6%

Behaviors	*n*	*Average behavior*
Average frequency of outdoor recreation		
Before 11 March	1,118	5.07 days per week
After 11 March	1,118	4.76 days per week
Average distance traveled to participate in outdoor recreation		
Before 11 March	877	3.50^a^
After 11 March	877	1.94^a^
Average distance traveled beyond roads during outdoor recreation		
Before11 March	728	4.77 miles
After 11 March	728	2.61 miles
Average outdoor recreation group size		
Before 11 March	940	5.61 persons
After 11 March	940	1.85 persons

a Scale: 1 = ‘0 to 2 miles’, 2 = ‘3 to 5 miles’, 3 = ‘6 to 15 miles’, 4 = ‘16 to 50 miles’, 5 = ‘≥ 50 miles’.

### ANOVA results

Across all four behaviors of interest, the magnitude of changes increased with community size (Table 3). Results of the four one-way ANOVAs and corresponding post hoc tests can be found in Table 3. Each ANOVA, with the exception of change in group size, yielded significant omnibus results—indicating significant differences among community classifications. Levene’s *F* test allows equality of variance to be assumed for all behaviors besides change in frequency. However, a subsequent Welch’s Test of Equality of Means indicates significant differences among at least two groups (Vaske 2008). Subsequent post hoc tests show that urban area residents decreased their frequency of outdoor recreation participation, distance travelled to participate in outdoor recreation and distance traveled beyond roads during outdoor recreation significantly more than rural residents. Urban area residents also decreased their distance travelled to participate in outdoor recreation significantly more than urban cluster residents.

**Table 3:** ANOVA results with post hoc tests on group differences

Behavior	Change	F-value	Levene statistic
Change in average frequency of outdoor recreation per week (*n* = 928)		3.16^a^	8.373^b^
Rural residents	−0.03 days^c^		
Urban cluster residents	−0.37 days		
Urban area residents	−0.52 days^d^		
Change in average distance traveled to participate in outdoor recreation (*n* = 862)		10.83^e^	2.362^f^
Rural residents	−1.31 scale points^c^		
Urban cluster residents	−1.45 scale points^c^		
Urban area residents	−1.83 scale points^d^,^g^		
Change in average distance traveled beyond roads during outdoor recreation (*n* = 642)		12.03^e^	1.313^f^
Rural residents	−1.52 miles^c^		
Urban cluster residents	−2.26 miles		
Urban area residents	−2.87 miles^d^		
Change in average outdoor recreation group size (*n* = 929)		1.553	1.959^f^
Rural residents	−3.01 persons		
Urban cluster residents	−3.63 persons		
Urban area residents	−4.38 persons		

a Statistically significant at a 95% confidence interval: at least two groups are significantly different.

b Equality of variances cannot be assumed.

c Statistically significantly different than urban area residents at a 95% confidence interval.

d Statistically significantly different than rural residents at a 95% confidence interval.

e Statistically significant at a 99.9% confidence interval: at least two groups are significantly different.

f Equality of variances can be assumed.

g Statistically significantly different than urban cluster residents at a 95% confidence interval.

## Discussion

While results of this study indicate that the behaviors of outdoor enthusiasts—despite the size of their community of residence—have changed in response to the COVID-19 pandemic, those from urban areas are significantly more impacted than those from urban clusters or rural areas. These results confirm the hypothesis of Samuelsson et al. (2020)—that travel and physical distancing restrictions are impacting recreation patterns of urban populations, who are generally bound by the tightest restrictions (Tufan and Kayaaslan 2020). While restrictions on outdoor recreation are necessary to thwart the pandemic, a number of unintended repercussions are likely to result from changes in outdoor recreation behavior. Specifically, diminished frequency of participation and distance travelled beyond roads have clear links to health and wellbeing consequences through limiting the attainment of recreational ecosystem services. Those who participate in outdoor recreation more frequently are likely to be in better health (Payne et al. 2005; Thomsen, Powell and Allen 2013). Additionally, backcountry recreation—beyond roads—offers a unique array of benefits distinct from those available through frontcountry recreation (Pohl, Borrie and Patterson 2000; Holland et al. 2018). Therefore, decreases in both frequency of participation and backcountry use among outdoor enthusiasts of all community classifications may have lasting unintended negative impacts on human health and wellbeing—though ecological wellbeing may benefit from this period of reduced outdoor recreation and travel (Rosenbloom and Markard 2020).

Additionally, decreases in distance travelled to participate in outdoor recreation—significantly greater among urban area residents—suggest that visitors are substituting more proximate recreational settings for more preferable, distant settings. This substitution behavior implies that outdoor enthusiasts are settling for sub-optimal experiences, or perhaps experiences they perceive to be sub-optimal in the planning stage of their experience (Brunson and Shelby 1993). It is possible that changes in behavior such as reduced travel distance may result in the discovery of acceptable, alternative local recreation areas (Tu et al. 2016). As posited by Salama (2020), this behavior may lead to a reconsideration of ‘home ranges’ and place identity for outdoor enthusiasts and the broader public. However, it also poses a current challenge to planners and local park and recreation practitioners attempting to encourage physical distancing.

### Implications and recommendations for planning and management

The importance of urban recreational ecosystem services is well-documented (see Zhang, Tan and Diehl 2017; Escobedo et al. 2019; Tan et al. 2020). In light of our research findings, we recommend that urban planners and public land managers work in concert to develop strategies for the facilitation of outdoor recreation for urban populations during the COVID-19 pandemic and ensure the provisioning of these recreational ecosystem services during this crisis and in future crises. One proposed strategy is the temporary transformation of roadways to pedestrian corridors, as done in Denver, CO and Oakland, CA (USA) (González 2020). This action alleviates dense use of pedestrian paths and increases residents’ capacity to practice physical distancing while still engaging in outdoor recreation activities like biking, running or walking.

Other direct management actions might also be employed. In parks with control points, we recommend that managers consider quotas to reduce the density of use during the pandemic while preventing the need for closures (see Manning 2011). Connecticut (USA) state parks have trialed this strategy by reducing parking capacity (Connecticut Department of Energy and Environmental Protection 2020). Additionally, timed entry systems might be employed, as proposed by managers at Rocky Mountain National Park (USA) (Blumhardt 2020). In urban, residential park settings alternative means of managing risk of infection are likely needed such as quotas within certain high use areas and trailheads or prohibiting use by non-local residents, as seen in the San Francisco (USA) metro area (Stienstra 2020). Indirect management measures should also be considered to manage outdoor recreation during the pandemic. Examples of such measures include the use of painted physical distancing reminders on multi-use paths in London (UK) (Samuelsson et al. 2020), signage with persuasive collectivist messaging encouraging responsible recreation practices and physical distancing in Oakland (USA) (Riggs 2020), or encouraging one-way traffic on trails in Boulder (USA) (O’Keefe 2020).

Finally, as observed by Salama (2020), planners and public land managers should consider the possibility of future pandemics in their recreation planning and design. Recommendations include designing spaces that adhere to spatial proximities that comply with physical distancing recommendations and creating more biophilic design elements in urban recreation settings (Salama 2020). Improved urban green infrastructure may prove as an effective means of increasing coping capacity and the provisioning of recreational ecosystem services (Palliwoda, Banzhaf and Priess 2020). In addition to underscoring the importance of park and outdoor recreation design, the COVID-19 pandemic—and our research of its impacts—highlights the value of access to open space in the midst of crisis. It is thus recommended that cities work to quantify this value and work to preserve remaining green space, as noted by Samuelsson et al. (2020).

### Limitations and future research

Primary among the limitations of this study is the self-reported, retrospective nature of the questions being asked. Retrospective reporting has been shown to bias results in other leisure contexts (Ito, Walker and Kono 2019). Demographic biases might also be impactful. It should be noted that the sample was not exclusively composed of US residents. While the study’s sample is overwhelmingly composed of non-Hispanic white (88.5%) individuals, this is in line with estimates of overall outdoor recreation participation (Askew and Walls 2019; Outdoor Foundation 2020). However, the sample is composed of a female majority (57.8%). This is not consistent with outdoor recreation participation at large (45.0%; Outdoor Foundation 2020). Other socio-demographic measures were not assessed, presenting the possibility of additional biases within the sample. The surveying of LNT listserv members also presents the possibility that the sample may be better educated about responsible outdoor recreation or disposed to following regulations than the larger outdoor recreation community.

Additionally, this study is limited by the lack of information concerning stay-at-home orders or other restrictions to travel enforced upon respondents. The timing enforcement, and adherence to these restrictions has varied across the USA and around the globe (Tufan and Kayaaslan 2020), and therefore restrictions may have varied impacts on the behaviors of respondents. The degree to which these necessary restrictions influenced outdoor recreation behavior is unknown. Finally, these results provide an understanding of a specific moment within a rapidly evolving pandemic. Therefore, the interpretation of these results should be considerate of the time when the data were collected—9th–11th April 2020, when there were more than 503,000 cases and 18,761 deaths in the U.S. attributed to COVID-19.

Based on our findings, three major areas of future research emerge. Primary among these research needs is quantifying the impact of the COVID-19 pandemic on the wellbeing of outdoor enthusiasts. Further inquiry concerning the consequences of these documented changes in behavior on mental and physical health outcomes is required to fully understand the pandemic’s impact on the outdoor enthusiast subculture (Rice et al. 2020). Second, future research is needed to understand exactly what aspects of the pandemic (e.g. agency guidance, regulations, closures, social norms, intrinsic motivations, etc.) have changed the recreational behaviors of outdoor enthusiasts. Urban planners and public land managers, alike, require information concerning the source of these recreation shifts to better facilitate recreation during the pandemic and following its conclusion. Finally, more research is merited concerning the shifts in these recreational behaviors during the remainder of the pandemic. Our results provide a ‘snapshot’ in time of recreation behaviors of outdoor enthusiasts, but in a rapidly evolving pandemic, additional samples would improve our understanding of how impacts are evolving as well.

## Conclusion

The COVID-19 pandemic has presented humankind with a tremendous challenge. With challenge comes stress and the need for reprieve. Our findings indicate that urban outdoor enthusiasts are disproportionately impacted by the recreational burden of the pandemic—reducing their participation, changing their recreation settings, and reducing backcountry recreation to greater degrees than residents of rural areas. Put another way, those most directly impacted by the pandemic (Stier, Berman and Bettencourt 2020) have also experienced the greatest impact on their ability to cope with the pandemic. Our findings suggest that planners and public land managers must provide additional consideration to the recreational capacity of urban residents during this crisis.

*Conflict of interest statement*. None declared. 
